# Experiences of Stigma Among Parents With Intellectual Disability: A Systematic Review and Thematic Synthesis

**DOI:** 10.1111/jar.70306

**Published:** 2026-09-29

**Authors:** Ben McFarlane, Zoey Robinson, David Curran, Stephanie Burns

**Affiliations:** ^1^ School of Psychology, Queen's University Belfast Belfast UK

**Keywords:** child protection, intellectual disability, parents, stigma, thematic synthesis

## Abstract

**Background:**

Parents with intellectual disabilities experience stigma across parenting, professional and child protection contexts, yet existing systematic reviews have focused primarily on support needs and interventions.

**Method:**

A systematic review and thematic synthesis was conducted to explore qualitative research on the lived experiences of stigma among parents with intellectual disabilities. Searches were conducted across CINAHL, MEDLINE, PsycINFO and Scopus. Twenty‐three studies met inclusion criteria.

**Results:**

Five analytical themes were identified: (1) Constructing parents as incompetent; (2) Regulating parenthood through surveillance and control; (3) Structural harm: Violence, coercion and family separation; (4) Living with stigma: identity, emotional impact and parenting experience; and (5) Negotiating and resisting stigma through support and agency.

**Conclusion:**

Stigma shaped parents' identities, relationships with professionals and family outcomes, while parents actively resisted deficit‐based assumptions. Findings highlight the need for accessible strengths‐based supports.

## Lay Summary

- Parents with intellectual disabilities are often judged as unable to care for their children before people fully understand what they can do or what support they may need.
- Parents described many difficult experiences, including: being closely watched by services, having less control over their decisions about their lives and families, and feeling upset from child protection involvement.
- Parents described parenting as an important and meaningful part of who they are. They talked about loving their children, standing up for themselves and their families, and challenging the belief that they are not capable parents.
- The findings show that parents with intellectual disabilities need information and support that is easy to understand, focuses on what they can do, and is provided by professionals who treat them with respect and support their independence.

## 1 Introduction

Stigma is a social process through which individuals or groups are judged and socially devalued (Goffman 1963). Moreover, stigma has been described as a process involving labelling, stereotyping, status loss and discrimination within contexts of unequal power (Link and Phelan 2001). Stigma can operate across interpersonal, institutional and structural levels, shaping access to resources, opportunities and wellbeing (Hatzenbuehler and Link 2014; Pescosolido and Martin 2015).

People with intellectual disabilities represent a population for whom stigma is unavoidable. Individuals with intellectual disabilities are frequently positioned within socially devalued roles, shaped by assumptions of dependency, incompetence and reduced autonomy (Scior 2011; Werner et al. 2012). Systematic reviews demonstrate that people with intellectual disabilities experience widespread stigma, including negative public attitudes, social distancing, and discrimination across multiple domains of life (Ali et al. 2012; Scior 2011). From early life through to adulthood, these processes are associated with social exclusion, reduced opportunities, poverty and poorer health and wellbeing outcomes (Emerson 2007; Emerson and Hatton 2007; Hatton and Emerson 2004). Further evidence indicates that individuals are often aware of these societal perceptions and may internalise aspects of stigma, shaping identity and self‐concept (Beart et al. 2005; Logeswaran et al. 2019; Monteleone and Forrester‐Jones 2017).

Within this context, the experiences of parents with intellectual disabilities have received increasing research attention. Individuals with intellectual disabilities express aspirations to parenthood, including desires to have children and provide care within family relationships (Mayes et al. 2006). Furthermore, parents with intellectual disabilities engage in everyday parenting practices and demonstrate parenting capacity, particularly when appropriate supports are available (Wade et al. 2008; Wilson et al. 2014). Additionally, parents often construct positive parenting identities alongside holding more negative views of their intellectual disability label (Shewan et al. 2014). These findings challenge deficit‐based assumptions and highlight the importance of recognising parents with intellectual disabilities as active participants in family life.

Nonetheless, parents with intellectual disabilities are disproportionately involved in child protection systems and are more likely to experience child removal than non‐disabled parents (Booth et al. 2005; McConnell and Llewellyn 2002; Slayter and Jensen 2019; Lightfoot and Dangle 2026). However, evidence regarding the actual risk of maltreatment is mixed, with some research suggesting that intellectual disability may function as a risk indicator rather than a direct causal factor (Slayter and Jensen 2019; Lightfoot and Slayter 2014). However, professional decision‐making within child protection systems may be influenced by assumptions regarding perceived risk and parental competency (Proctor and Azar 2013).

These findings indicate that stigma plays a significant role in shaping the parenting experiences of individuals with intellectual disabilities. Stigma may be experienced in interactions with others, within professional and service contexts, and in the broader social and structural conditions that influence parenting outcomes. Such experiences can impact parental identity, confidence, and engagement with support services, while also contributing to unequal outcomes, including disproportionate involvement in child protection systems (Jahoda et al. 2010; Lightfoot and Dangle 2026; Slayter and Jensen 2019). Notwithstanding, parents actively resist stigmatising narratives and assert their identities as parents (Pacheco and McConnell 2017).

There is a growing body of literature relating to parents with intellectual disabilities; however, much of this research has focused on parenting capacity, risk and intervention outcomes, often framing parenting as a deficit (Llewellyn and Hindmarsh 2015; Slayter and Jensen 2019). This emphasis reflects a broader tendency to conceptualise parents as inherently vulnerable or problematic, with comparatively limited attention given to their subjective experiences.

A recent review within the wider intellectual disability literature demonstrates that stigma is internalised and negotiated in ways that shape identity, self‐concept and social behaviour (Logeswaran et al. 2019). The authors describe that individuals are often aware of the negative societal meanings attached to the intellectual disability label and may distance themselves from it, or experience shame, powerlessness and exclusion.

Existing reviews have primarily focused on support needs and intervention effectiveness (Koolen et al. 2020; Wilson et al. 2014), often privileging professional perspectives. A recent scoping review by Morris et al. (2026) explored the literature on parenting experiences among adults with intellectual disabilities and identified ableism as a prevalent barrier across institutional and interpersonal contexts. As a scoping review, it did not include appraisal of methodological quality and was not designed to provide an in‐depth interpretive synthesis of stigma as a central construct. Given the central role of stigma discussed in shaping experiences and outcomes, a focused synthesis is required to explore how stigma is experienced, interpreted and negotiated within parenting contexts.

This current systematic review and thematic synthesis aims to address this gap by answering the research question: ‘What are the lived experiences of stigma described by parents with intellectual disabilities?’ Specifically, the review aims to (1) identify and synthesise qualitative research exploring these experiences, (2) examine the forms, sources and contexts of stigma, including interpersonal, institutional and societal levels, (3) explore how stigma influences parents' self‐perceptions, parenting identity and sense of competence, (4) examine the impact of stigma on relationships with professionals and engagement with support services; and (5) identify coping strategies, responses and acts of resistance described by parents.

## 2 Method

Throughout the development of the protocol for this systematic review, guidance on the Preferred Reporting Items for Systematic Reviews and Meta‐Analyses (PRISMA) checklist (Page et al. 2021) was followed. In addition, the RETREAT framework (Booth et al. 2018) was used to guide the selection of thematic synthesis (Thomas and Harden 2008) as the most appropriate qualitative evidence synthesis method. Further to this, guidance regarding data extraction (Noyes et al. 2018) was utilised throughout. A systematic review protocol was registered with Prospero: registration number CRD420251149790.

### 2.1 Search Strategy

The PICo (Population, phenomenon of Interest, Context; Stern et al. 2014) was chosen to develop the research question and subsequent search strategy. PICo was considered most appropriate due to the specific qualitative topic (i.e., experiences of stigma) of this review (Stern et al. 2014). The databases CINAHL, MEDLINE, PsycINFO and Scopus were systematically searched. The search was completed in September 2025 using the following search terms: (‘intellectual disabilit*’ OR ‘learning disabilit*’ OR ‘mental retardation’ OR ‘intellectual impairment’ OR ‘intellectual deficit’ OR ‘cognitive impairment’ OR ‘cognitive deficit’ OR ‘mental deficiency’) AND (parent* OR mother* OR father* OR guardian*) AND (stigma* OR discriminat* OR prejudice OR marginali* OR stereotyp* OR attitude* OR label* OR devalu* or isolate*) AND (qualitative OR interview* OR ‘focus group*’ OR thematic OR grounded OR ethnograph* OR ‘case study’ OR phenomenology* OR narrative). This search string was adapted specifically for each database. In line with qualitative evidence synthesis methodology, these search terms were collaboratively developed by the authors, no limiters were used, and qualitative methodological terms were included to facilitate retrieval of relevant studies (Booth 2016). Additionally, backward reference searches were conducted to identify relevant studies where experiences of stigma may not have been explicit in titles, abstracts or indexing terms, in adherence with qualitative evidence synthesis (Booth 2016).

### 2.2 Inclusion and Exclusion Criteria

Studies were eligible for inclusion if they employed qualitative methodologies and reported first‐person experiential data from parents with an intellectual disability. In line with the PICo framework, the population of interest comprised parents (aged 18 years and over) with an intellectual disability. Studies were included where the phenomenon of interest related to experiences of stigma, including discrimination, prejudice, marginalisation or related constructs, in the context of the parenting role. No restrictions were placed on context, and studies conducted across home, community, and professional service settings were considered eligible. Studies using mixed‐methods designs were included where qualitative data could be clearly identified and extracted. Data derived directly from parents with intellectual disabilities and authors' interpretive text regarding these data were included in the synthesis. Where studies included additional perspectives, only data relating directly to parents' experiences were extracted and synthesised.

Studies were excluded if they (1) did not include participants with an intellectual disability, or where this could not be clearly established, (2) did not include participants describing their lived experience of parenting, (3) focused on third‐party perspectives (e.g., professionals, caregivers or children) without including first‐person parental accounts, (4) did not address experiences of stigma or related constructs in relation to parenting or (5) employed exclusively quantitative methodologies. Review articles (e.g., systematic or scoping reviews) were also excluded. In line with the review protocol, peer‐reviewed studies and grey literature were included. Studies were required to be written in English, and published between 2010 and 2025 to capture contemporary research aligned with international rights‐based approaches to disability following the UN Convention on the Rights of Persons with Disabilities (United Nations 2006).

### 2.3 Study Identification

A PRISMA flow diagram outlining the study identification process is presented in Figure 1. Search results identified 2594 articles which were imported into Covidence, where 782 duplicate records were removed. A further seven duplicates were identified and removed during the screening process. Title and abstract screening were conducted by the first reviewer (BMcF) for all records. A second reviewer (ZR) independently screened 20% of titles and abstracts using Covidence. An inclusive approach was adopted at this stage to ensure that potentially relevant studies were retained. Inter‐rater agreement was 99.7% (Cohen's *κ* = 0.89); one disagreement was resolved through discussion. Full‐text screening was conducted independently by both reviewers (BMcF and ZR) for all remaining studies (*n* = 36). Initial inter‐rater agreement was 75% (Cohen's *κ* = 0.45), with nine disagreements identified. Eight were resolved through discussion between reviewers, and one disagreement was resolved through consultation with a third reviewer (SB). Following database screening, backward reference searching was conducted on 16 included papers, resulting in the identification of an additional seven studies. In total, 23 studies met the inclusion criteria and were included in the final synthesis.

**FIGURE 1:**
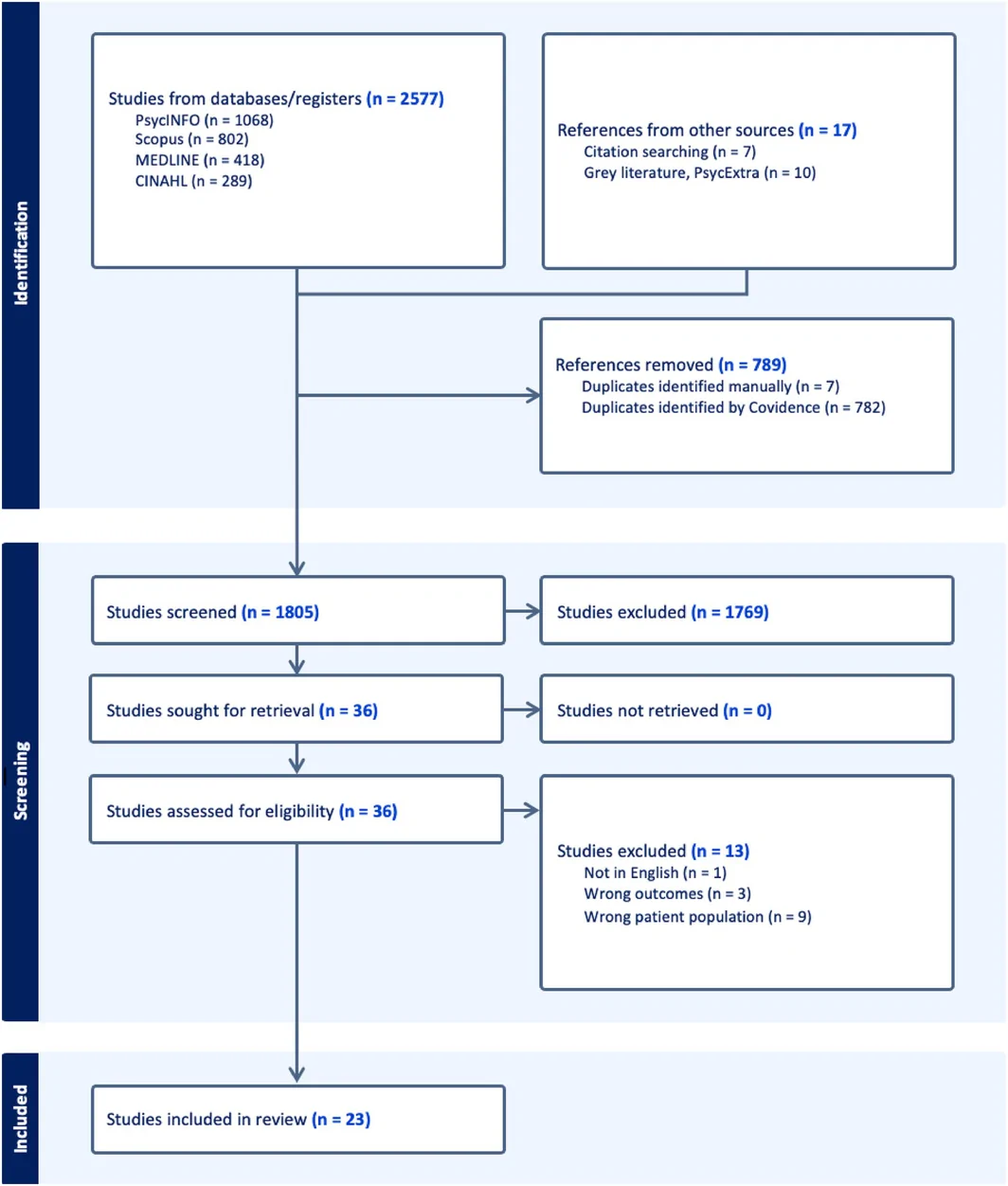
PRISMA (Page et al. 2021) flowchart for selection of studies.

### 2.4 Data Extraction

Data extraction was informed by guidance from Noyes et al. (2018). A structured data extraction form was developed and piloted to ensure consistency. Extracted information included study characteristics (authors, year, country), study aims, setting, participant characteristics (including type of intellectual disability, age, gender, parenting status and ethnicity), sampling approach, data collection methods and data analysis approach, see Table 1. In line with thematic synthesis methodology (Thomas and Harden 2008), the results sections of included studies were extracted in full and subjected to line‐by‐line coding, including both participant quotations and authors' interpretative accounts. Data extraction was conducted by the first reviewer (BMcF) and reviewed in discussion with academic supervisors to enhance rigour and consistency.

**TABLE 1:** Study characteristics.

Study identification	Country	Aims of study	Participant characteristics (diagnosis, functioning, age, gender, parenting status)	Sampling approach	Data collection methods	Data analysis method
Atkin and Kroese (2022)	United Kingdom	To explore parents and independent advocates experiences of child protection proceedings (CPP).	Type of intellectual disability: 4 mothers with intellectual disabilities (noted as having comprehension and communication difficulties). Age: 20s to 30s (for parent participants). Gender: Female (all 4 parents and all 4 advocates). Parenting status: Collectively the four mothers had 17 children. Their status varied: one mother had four children removed and adopted; one had two children in long‐term foster care and one adopted; one had three in foster care and two in her care; and one mother and her partner had kept all three of their children following proceedings. Ethnicity: White British (3), Pakistani British (1)	Purposive	Semi‐structured interviews conducted using a schedule developed in consultation with a parent with intellectual disabilities who had prior experience of CPP.	Interpretative Phenomenological Analysis
Ćwirynkało and Parchomiuk (2023)	Poland	To explore fathers with intellectual disabilities experiences of social support.	Type of intellectual disability: 20 fathers (14 with mild and 6 with moderate intellectual disabilities). Age: 21 to 54 years. Gender: Male. Parenting status: All participants had contact with their children; 12 held full parental rights, while 8 had rights limited or terminated. Number of children per father ranged from 1 to 10. Ethnicity: Caucasian and Polish (20)	Purposive, using a snowball sampling method.	Semi‐structured interviews	Interpretative Phenomenological Analysis
Ćwirynkało and Parchomiuk (2024)	Poland	To explore fathers with intellectual disabilities experience of child protection proceedings and the meanings they attributed to them.	Type of intellectual disability: 14 fathers (8 with mild and 6 with moderate intellectual disabilities). Age: 21 to 54 years. Gender: Male. Parenting status: All participants had a history of being involved in child protection proceedings. At the time of the study, nine fathers did not exercise full parental responsibilities or had limited rights for at least one of their children. Ethnicity: Not reported.	Purposive	Semi‐structured interviews	Interpretative Phenomenological Analysis
Franklin et al. (2022)	United Kingdom	To explore parenting experiences among people with intellectual disabilities, focusing on stigma.	Type of intellectual disability: 22 participants (17 mothers and 5 fathers) who self‐identified as having learning disabilities. Age: 26 to 61 years (4 participants unreported). Gender: 17 females, 5 males. Parenting status: 15 had experienced temporary or long‐term child removal, 13 were living apart from their children, and 9 were living with their children at the time of the study. Ethnicity: White British (17), Black British (1), Indian (1), British Pakistani (1), Black British/Caribbean (1), mixed White & Black Caribbean (1)	Purposeful recruitment via seven different self‐advocacy groups.	Semi‐structured interviews co‐facilitated by learning‐disabled co‐researchers and supported by a ‘writing mentor’	Thematic Analysis
Gould and Dodd (2014)	United Kingdom	To explore mothers experiences of child removal and its impact.	Type of intellectual disability: Nine mothers with mild learning disabilities. Age: 21 to 39 years. Gender: Female. Parenting status: All participants had at least one child removed from their care at least 1 year prior to the interview. At the time of the study, three of the mothers had other children currently living at home with them. Ethnicity: Not reported.	Purposive	Semi‐structured interviews using a schedule developed in consultation with clinical experts. The guide used simple language and referenced significant life events to facilitate understanding for participants.	Interpretative Phenomenological Analysis
Höglund and Larsson (2013)	Sweden	To explore the experiences of pregnancy and childbirth among women with intellectual disabilities.	Type of intellectual disability: Ten women with a formal diagnosis of intellectual disability (all were known to formal intellectual disability services). Age: 21 to 41 years. Gender: Female. Parenting status: All had given birth between 2004 and 2010. They had between 1 and 7 children (Mean = 2.2). Five women had custody of their children, while the other five were not the primary caregivers (children were in foster or residential care), though one of these five retained custody of her younger children. Ethnicity: Not reported.	Modified snowball sampling.	Qualitative semi‐structured interviews using a topic guide developed by a multidisciplinary team. Each woman was interviewed twice over two consecutive days to establish rapport and allow them to expand upon or change their stories. Interviews were audio‐recorded and transcribed verbatim.	Content analysis. This involved an iterative process of dividing text into meaning units, condensing them into codes and grouping them into 14 sub‐categories and four main categories.
Hevesi and Theodore (2024)	United Kingdom	To explore mothers' experience of developing emotional relationships with their children.	Type of intellectual disability: 10 mothers who self‐identified as having learning disabilities (either diagnosed or inferred through attendance at advocacy groups). Age: 30 to 49 years (Mean = 40.75). Gender: Female. Parenting status: All were actively parenting at least one child; collectively they had 30 children ranging from 3 to 20 years old. All participants lived independently. Ethnicity: White British (5), White Other (1), Black British (1), Bangladeshi (1), Pakistani British (1), Other/Mixed (1).	Purposeful recruitment via advocacy groups across the UK.	Semi‐structured qualitative interviews using an interview guide developed in consultation with a peer support group of parents with learning disabilities.	Thematic Analysis
MacIntyre and Stewart (2012)	Scotland	To identify the needs of parents with intellectual disabilities and explore the role of advocacy meeting these needs.	Type of intellectual disability: Five parents who formally identified as having a learning disability and were assessed by an advocacy organisation as having the capacity to provide informed consent. Age: Between 24 and 45 years old. Gender: All five parent participants were female (mothers). Parenting status: All participants were originally referred for advocacy following the start of child protection proceedings; at the time of the study, the majority of their children were in foster care, accommodated by the local authority, or in kinship care. Ethnicity: Not reported.	A mixed‐method pilot study approach was used; mothers were randomly selected from a group of 11 eligible individuals currently receiving support from a single voluntary advocacy organisation	The study utilised in‐depth interviews with the parents, their assigned advocates, and key stakeholders (social workers and network coordinators), supplemented by a survey of 94 local organisations (24% return rate)	The researchers used both inductive and deductive approaches to code and analyse the emerging themes
Malouf et al. (2017)	United Kingdom	To explore experiences of women with intellectual disabilities in relation to pregnancy, childbirth, maternity services and support needs.	Type of intellectual disability: Nine women; eight were reported by support workers to have mild‐to‐moderate learning disabilities, and one was reported by a family member to have severe learning disabilities. Age: Between 25 and 39 years (mean and median of 30 years). Gender: All nine participants were female. Parenting status: Varied; at the time of interview, one was pregnant, and eight were mothers with between one and five children. Two women had experienced child removal from their care. Ethnicity: White (9).	Convenience	Semi‐structured interviews. The process included the use of easy‐read information materials and illustrative photos or video clips to facilitate communication.	Interpretative Phenomenological Analysis
McCarthy (2019)	United Kingdom	To explore how mothers with intellectual disabilities experience parenting in the context of domestic violence.	Type of intellectual disability: Six mothers with mild learning disabilities (recruited via professional contacts in learning disability services). Age: 23 to 41 years (Mean not specified; individual ages: 23, 23, 33, 36, 39, 41). Gender: Female. Parenting status: The six mothers had 13 children; 10 of these children had been removed from their care while the mothers were experiencing domestic violence. Ethnicity: White British (4), Bangladeshi (2).	Purposive sampling.	Semi‐structured interviews	Interpretative Phenomenological Analysis
Pacheco and McConnell (2017)	Canada	To explore the experiences of mothers with intellectual disability from ethnocultural communities in Canada.	Type of intellectual disability: Eight mothers with a formal diagnosis of intellectual disability. Age: 21 to 50 years. Gender: Female. Parenting status: Their children's status ranged from living at home to foster care and adoption. Ethnicity: Ethnocultural heritages reported, Indian (1), Portuguese (1), Polish (1), Trinidadian (1), Chinese (1), Aboriginal Metis (1), Aboriginal Cree (1), Trini‐Indian (1).	Purposive	Narrative methods involving 33 in‐depth interviews over a 2‐year period (3 to 7 per woman). Family photographs were used to develop rapport and open the conversation.	Narrative analysis
Pytlowana and Kroese (2021)	United Kingdom	To explore fathers with intellectual disabilities experiences of child removal through the English Family Court system.	Type of intellectual disability: Five fathers with mild to moderate intellectual disabilities (reported by referring professionals). Age: Range not explicitly stated to preserve anonymity, but participants included both younger fathers and grandfathers. Gender: Male. Parenting status: All participants had at least one biological child removed through the court system at least 1 year prior. Ethnicity: Not reported to protect anonymity.	Purposive	Semi‐structured interviews using ice‐breaker questions and a visual prompt (three concentric circles) to map helpful and unhelpful people in their lives.	Interpretative Phenomenological Analysis
Sheerin et al. (2013)	Republic of Ireland	To explore mothers with intellectual disabilities interactions with Child and Family Services in Ireland.	Type of intellectual disability: Four women in the mild–moderate range of intellectual disability. Age: 19 to 35 years. Gender: Female. Parenting status: Participants included mothers in the postnatal period and early motherhood (child under 5). One mother's child was currently in foster care. Ethnicity: Not reported.	Self‐selecting/Snowballing sample.	Semi‐structured interviews using a flexible guide developed through literature review and consultation with self‐advocates.	Thematic analysis
Starke (2010)	Sweden	To explore the experiences of mothers with intellectual disabilities interaction with support practitioners.	Type of intellectual disability: Seven mothers with an intellectual disability. Age: Between 24 and 42 years old. Gender: Female. Parenting status: The seven mothers had 14 children between them (aged 2 to 15). Living arrangements varied: one was married, others lived alone with children, or with extended family, and two had children in out‐of‐home care. Ethnicity: Not reported.	Exploratory study using participants contacted through adult rehabilitation centres.	Semi‐structured interviews	Textual analysis
Stefánsdóttir et al. (2023)	Iceland	To explore how a family led by parents with intellectual disabilities responded to child protection intervention and maintained family integrity.	Type of intellectual disability: One couple (Anna and Tómas) both identified as having learning disabilities. Age: Both parents were in their 30s at the time of the study. Gender: One female (mother) and one male (father). Parenting status: The couple had raised three children over a dozen years and were expecting their fourth child at the time of writing; they successfully maintained custody throughout. Ethnicity: Not reported.	An in‐depth qualitative case study of a single couple.	The study used a longitudinal approach including interviews (with parents, family, advocates and legal professionals), document analysis (child protection case files and intervention plans), participant observation in the home and digital communication (texting, video calls).	Grounded theory
Strnadová et al. (2019)	Czech Republic	To explore how women with intellectual disabilities develop a maternal identity and what impacts this process.	Type of intellectual disability: 19 women with a diagnosis of intellectual disabilities (described as having mild to moderate levels). Age: 23 to 48 years (Mean = 31.2). Gender: Female. Parenting status: All participants had given birth; collectively they had 31 children (aged 6 weeks to 27 years). Only eight women had full custody of all their children; others had children removed or were ‘non‐residential’ mothers. Ethnicity: Not reported.	Purposive	Semi‐structured interviews using an interview protocol divided into four parts. A follow‐up interview (with 13 of the original 19 mothers) was conducted to verify the emerging core theoretical concept.	Grounded theory
Sumskiene and Gevorgianiene (2025)	Lithuania	To explore the experiences and circumstances around child removal among mothers with intellectual disabilities.	Type of intellectual disability: 12 mothers with a diagnosed intellectual disability. Age: Between 28 and 55 years old. Gender: Female. Parenting status: All participants had experienced the loss of custody of at least one child. The age of the children at the time of removal ranged from birth to 12 years. Ethnicity: Not reported.	Convenience	Semi‐structured interviews	Thematic analysis
Symonds et al. (2021)	England	To explore fathers with a intellectual disabilities experiences of fatherhood and support.	Type of intellectual disability: Eight fathers who either self‐identified as having a learning disability or had direct experience of using learning disability services. Age: Between 26 and 61 years (Mean = 42). Gender: Male. Parenting status: All were birth fathers who had been in a relationship with the mother at the time of the birth. Their children ranged in age from 16 months to 34 years. At the time of the study, four fathers lived with their children and four lived separately. Ethnicity: White British (6), Black British (1), Pakistani British (1).	Purposive	Semi‐structured interviews	Thematic analysis
Tahir and Cobigo (2025)	Canada	The explore barriers and facilitators to positive outcomes for parents with intellectual disabilities involved in child protection.	Type of intellectual disability: 13 parents who had either a formal diagnosis of an intellectual disability or were in receipt of developmental support services. Age: Between 29 and 43 years old. Gender: 12 mothers and one father. Parenting status: All participants had a history of being involved with child protection. At the time of the study, four parents had experienced the permanent loss of custody, while nine had retained custody or had ongoing cases. Ethnicity: White (3), Black (3), Arab (2), Indigenous and White (2), Unavailable (3).	Inclusive research design utilising recruitment via local and regional developmental support agencies.	Semi‐structured interviews (available in both English and French) utilising Easy‐to‐read materials and introduction by the co‐researcher.	Thematic analysis
Theodore et al. (2018)	United Kingdom	To explore the experiences of parents with intellectual disabilities through collaboratively collected personal accounts.	Type of intellectual disability: Eight participants who self‐identified as having a learning disability. Age: 31 to 61 years. Gender: Five mothers and three fathers. Parenting status: Collectively they had 22 children (aged 2 to 29 years); seven parents had at least one minor child. Six parents had experienced separation from their children; three were currently living with at least some of their children. Ethnicity: White British (5), British Pakistani (1), Black British/Caribbean (1), Mixed Caribbean/Asian (1).	Purposive	Semi‐structured interviews led by learning‐disabled principal researchers and supported by a ‘writing mentor’.	Thematic analysis
Wilmots and Theodore (2025)	United Kingdom	To explore fathers with intellectual disabilities experiences of developing attachment with their children.	Type of intellectual disability: 11 fathers who self‐identified as having a learning disability (3 had a formal diagnosis, 8 self‐identified). Age: 28 to 54 years (Mean = 39.45). Gender: Male. Parenting status: Participants were actively parenting children aged 25 or younger (currently or in the past). Six fathers were currently actively parenting, and four were co‐parenting. Ethnicity: White British (7), Black British (3), British Pakistani (1).	Purposive	Semi‐structured interviews averaging 57 min.	Interpretative Phenomenological Analysis
Wołowicz and Ćwirynkało (2025)	Poland	To explore experiences of parents with intellectual disabilities regarding violence and restrictions on parenting rights.	Type of intellectual disability: 27 parents with mild to severe intellectual disabilities. Age: Between 21 and 54 years old (Mean = 37.41). Gender: 16 mothers and 11 fathers. Parenting status: All participants had contact with their children; 10 held full parental rights, while 16 had limited or no parental rights (with children usually placed in foster or kinship care). Ethnicity: Not reported.	Purposive	Individual, semi‐structured interviews lasting approximately 50 to 90 min. Researchers used accessible language and conducted interviews in locations where participants felt comfortable.	Thematic Analysis
Wos and Baczała (2021)	Poland	To explore mothers with intellectual disabilities everyday experiences, challenges and support.	Type of intellectual disability: Seven mothers with mild to moderate intellectual disabilities. Age: Between 22 and 43 years old. Gender: Female. Parenting status: All participants held full parental rights and lived with their children at the time of the study. Ethnicity: Not reported.	Purposeful	The photovoice method was used; participants took series of photographs (an average of 10 per person) over 7 days based on the prompt ‘What does my parenthood look like?’ followed by individual in‐depth interviews to interpret the images.	Thematic data analysis

### 2.5 Quality Assessment

The methodological quality of included studies was assessed using the CASP qualitative checklist (Critical Appraisal Skills Programme 2024). The CASP checklist comprises 10 items assessing the validity, methodological rigour and relevance of qualitative research. All included studies were appraised by the first reviewer (BMcF). A second reviewer (ZR) independently appraised 20% of studies to enhance reliability. Agreement between reviewers was 100%, and no discrepancies required resolution. Appraisal ratings of ‘yes’, ‘no’ and ‘can't tell’ were applied in line with CASP guidance, see Table 2. In line with recommendations for qualitative evidence synthesis, studies were not excluded based on quality appraisal, findings were used to inform the interpretation of results.

**TABLE 2:** CASP checklist quality criteria.

Study author & year	Q. 1, clear statement for aims?	Q. 2, appropriate methodology?	Q. 3, appropriate design?	Q. 4, appropriate recruitment strategy?	Q. 5, appropriate data collection?	Q. 6, research‐participant relationship considered?	Q. 7, ethical issues considered?	Q. 8, rigorous data analysis?	Q. 9, clear findings presented?	Q. 10, research value demonstrated?
Atkin and Kroese (2022)	Yes	Yes	Yes	Yes	Yes	Yes	Yes	Yes	Yes	Yes
Ćwirynkało and Parchomiuk (2023)	Yes	Yes	Yes	Yes	Yes	Can't tell	Yes	Yes	Yes	Yes
Ćwirynkało and Parchomiuk (2024)	Yes	Yes	Yes	Yes	Yes	Can't tell	Yes	Yes	Yes	Yes
Franklin et al. (2022)	Yes	Yes	Yes	Yes	Yes	Yes	Yes	Yes	Yes	Yes
Gould and Dodd (2014)	Yes	Yes	Yes	Yes	Yes	Can't tell	Yes	Yes	Yes	Yes
Hevesi and Theodore (2024)	Yes	Yes	Yes	Yes	Yes	Can't tell	Yes	Yes	Yes	Yes
Höglund and Larsson (2013)	Yes	Yes	Yes	Yes	Yes	Yes	Yes	Yes	Yes	Yes
MacIntyre and Stewart (2012)	Yes	Yes	Yes	Yes	Yes	Can't tell	Yes	Yes	Yes	Yes
Malouf et al. (2017)	Yes	Yes	Yes	Yes	Yes	Can't tell	Yes	Yes	Yes	Yes
McCarthy (2019)	Yes	Yes	Yes	Yes	Yes	Can't tell	Yes	Yes	Yes	Yes
Pacheco and McConnell (2017)	Yes	Yes	Yes	Yes	Yes	Can't tell	Yes	Yes	Yes	Yes
Pytlowana and Kroese (2021)	Yes	Yes	Yes	Yes	Yes	Yes	Yes	Yes	Yes	Yes
Sheerin et al. (2013)	Yes	Yes	Yes	Yes	Yes	Can't tell	Yes	Can't tell	Yes	Yes
Starke (2010)	Yes	Yes	Yes	Yes	Yes	Can't tell	Yes	Can't tell	Yes	Yes
Stefánsdóttir et al. (2023)	Yes	Yes	Yes	Yes	Yes	Yes	Yes	Yes	Yes	Yes
Strnadová et al. (2019)	Yes	Yes	Yes	Yes	Yes	Yes	Yes	Yes	Yes	Yes
Sumskiene and Gevorgianiene (2025)	Yes	Yes	Yes	Yes	Yes	Yes	Yes	Yes	Yes	Yes
Symonds et al. (2021)	Yes	Yes	Yes	Yes	Yes	Yes	Yes	Yes	Yes	Yes
Tahir and Cobigo (2025)	Yes	Yes	Yes	Yes	Yes	Yes	Yes	Yes	Yes	Yes
Theodore et al. (2018)	Yes	Yes	Yes	Yes	Yes	Yes	Yes	Yes	Yes	Yes
Wilmots and Theodore (2025)	Yes	Yes	Yes	Yes	Yes	Yes	Yes	Yes	Yes	Yes
Wołowicz and Ćwirynkało (2025)	Yes	Yes	Yes	Yes	Yes	Yes	Yes	Yes	Yes	Yes
Wos and Baczała (2021)	Yes	Yes	Yes	Yes	Yes	Yes	Yes	Yes	Yes	Yes

*Note:* Critical Appraisal Skills Programme. Q1–Q10 correspond to the 10 CASP qualitative checklist items (Critical Appraisal Skills Programme 2024). Responses indicate whether criteria were met ‘Yes’, ‘No’ or ‘Can't tell’.

### 2.6 Data Synthesis

Data were synthesised using thematic synthesis (Thomas and Harden 2008). This approach was selected using the RETREAT framework (Booth et al. 2018) as an appropriate method for addressing the interpretive aims of the review. The synthesis followed three stages. First, the full results sections of included studies were imported into NVivo and subjected to inductive line‐by‐line coding, including both participant quotations and authors' interpretative accounts. Second, codes were organised into descriptive themes that remained closely aligned with the content of the original studies. Third, descriptive themes were further interpreted to generate analytical themes that synthesise the findings of the included studies. The process of theme development was iterative, with codes and themes refined through ongoing comparison across studies. Emerging themes were reviewed and discussed with academic supervisors to enhance the credibility and coherence of the analysis. Reflexive consideration was supported through the maintenance of a reflective log throughout the analytic process. The analysis was informed by a critical understanding of stigma as structurally produced, which shaped how accounts of professional involvement were interpreted.

## 3 Results

### 3.1 Study Characteristics

A total of 23 studies met the inclusion criteria, with 16 identified through database searches and seven through backward reference searching. As shown in Table 1, the included studies were conducted across a range of countries, including Canada, Czech Republic, Iceland, Lithuania, Poland, Republic of Ireland, Sweden and the United Kingdom. Sample sizes ranged from one couple to 27 parents. Participant ages ranged from 19 to 61 years. Combined reported sample sizes totalled 240 participants, including 161 mothers and 79 fathers. Thirteen studies included mother‐only samples, five included father‐only samples, and five included both mothers and fathers. Ethnicity was inconsistently reported, with explicit information available in 11 of 23 studies. Where reported, participants were predominantly White or White British, with representation from Black, South Asian, Arab, Indigenous, mixed‐ethnicity and other ethnocultural backgrounds (see Table 1).

Methodological approaches included interpretative phenomenological analysis, thematic analysis, grounded theory, photovoice and longitudinal narrative methods. Several studies used inclusive research approaches, involving adults with intellectual disabilities in co‐production aspects of study design or data collection.

The methodological quality of included studies was generally high according to the CASP checklist (see Table 2). All studies provided clear statements of aims and findings. Stronger studies demonstrated clear recruitment strategies, detailed analytic procedures and accessible approaches to participation. The most common area of ambiguity concerned reflexivity, with several studies providing limited detail regarding the researcher‐participant relationship or the management of power dynamics during data collection. No studies were excluded based on quality appraisal.

### 3.2 Themes

Following the thematic synthesis approach outlined by Thomas and Harden (2008), the findings were synthesised into five analytical themes and 12 descriptive subthemes. Together, these themes describe how stigma was constructed, enacted, internalised and resisted in the lives of parents with intellectual disabilities, see Table 3.

**TABLE 3:** Analytic themes and descriptive subthemes developed from thematic synthesis.

Analytic themes	Descriptive sub‐themes & (No. of papers contributing)
1.0 Constructing parents as incompetent.	1.1 Negative biases (21). 1.2 Being judged and devalued (22).
2.0 Regulating parenthood through surveillance and control.	2.1 Monitoring and system control (22). 2.2 Systemic Barriers (21).
3.0 Structural Harm: Violence, Coercion and Family Separation.	3.1 Family conflict and power imbalances (22). 3.2 Child removal, loss and threat (22). 3.3 Violence, coercion and structural harm (13).
4.0 Living with stigma: identity, emotional impact, and parenting experience.	4.1 Parenting identity, meaning, and emotional experience (20). 4.2 Parenting practices, relationships, and child experience (21).
5.0 Negotiating and resisting stigma through support and agency.	5.1 Support systems: Family, professionals, and resources (21). 5.2 Advocacy, agency, and resistance (21). 5.3 Navigating support, learning, and adaptation (20).

#### 3.2.1 Theme 1: Constructing Parents as Incompetent

This analytic theme contains two sub‐themes and describes how parents with intellectual disabilities are often positioned as inherently inadequate, requiring ongoing proof of competence of their parenting skills in ways not expected of the general population. Their lived experiences are positioned within a dominant narrative of parental incompetency.

##### 3.2.1.1 Subtheme 1.1: Negative Biases

This sub‐theme captures the consistent descriptions of assumed incompetent parenting across 21 included studies of parents with intellectual disability by professionals and others. These negative evaluations of parenting ability are in the context of the individual's diagnostic label of intellectual disability, are often made quickly, and prior to formal assessments being conducted. Lisa described the immediacy of this judgement, ‘They say learning disability can't look after a child and they just assume straight away that you can't look after your child’ (Theodore et al. 2018). Furthermore, Marie described how professional doubts repeatedly returned to her diagnosis, ‘They don't think I can cope, because I have got a learning disability… it always came back to that’ (Franklin et al. 2022).

Parents often describe a pressure to demonstrate their competence and emphasise responsibility, for example, Ken reported, ‘I have done two parenting courses, I have passed both parenting courses’. (Pytlowana and Kroese 2021). However, participants are met with impossible standards and low expectations from others. In Malouf et al. (2017) Morgan expressed, ‘For the last 8 months we've had to prove ourselves really. And we shouldn't have had to’. Overall, these experiences of stigma at a systemic and relational level suggest a wider deficit parenting narrative.

##### 3.2.1.2 Subtheme 1.2: Being Judged and Devalued

The following sub‐theme was applicable across 22 included studies. Parents described experiences of criticism, blame, and discriminatory treatment from professionals, for example, Patricia reported ‘It's just them dictating and I don't like that … ‘You're not a good mother. You're not this. You're not that …’ (Theodore et al. 2018). A range of interpersonal experiences, such as, feeling devalued and treated unfairly were highlighted throughout. As Dawn described feeling excluded from conversations about her family, ‘Social Services talked to them [other professionals] more than they talked to me… when they did talk, they treat us like a two‐year‐old, talking down to us…’ (Franklin et al. 2022). Tod also explained emotional challenges, ‘I just felt angry coz at the end of the day, we told them what we went through school and how we thought it would be different when we become adults and it's not, it's exactly the same’. (Pytlowana and Kroese 2021). An array of emotional consequences was described from participants, including, distress, fear and shame. These experiences of improper treatment frequently left parents feeling infantilised and misunderstood, regardless of their knowledge of their own children. As Debbie states, ‘Treat me like a mum, not a child. Like an adult’ (Gould and Dodd 2014).

#### 3.2.2 Theme 2: Regulating Parenthood Through Surveillance and Control

This analytic theme describes how stigma operates within systems that monitor, assess and constrain parents, often through inaccessible and exclusionary processes. Professional involvement was frequently experienced as surveillance rather than support, particularly where services were perceived as focused on risk.

##### 3.2.2.1 Subtheme 2.1: Monitoring and System Control

This sub‐theme explores parents' experiences, across 22 studies of living under scrutiny from professionals. Support was often experienced as intrusive or threatening, particularly where daily parenting decisions were perceived to be assessed for evidence of failure. Mary described the intensity of professional involvement shortly after birth: ‘…so all the social workers got involved and were always hovering around me so… [This started] … like 2 days after I had [baby]…they were all around me…’ (Sheerin et al. 2013). Parents described feeling controlled by services and pressured to comply with decisions. In Ćwirynkało and Parchomiuk 2023, Father 3 stated ‘I told her that […] we do not want a family assistant […] such an assistant just looks around, checks your fridge and look down on you!’. This fragility of professional relationships was exacerbated by reports of false accusations, risk labelling and parents often feeling ignored. Vita reported ‘Maybe the most difficult thing was the visits of the social worker. She used to come and check and comment on everything… I think there should have been fewer comments. It was very nerve‐wracking, somehow. Like hammering my head’. (Sumskiene and Gevorgianiene 2025). This perceived scrutiny was associated with increased anxiety of service involvement, particularly regarding child protection; this is contextually important for the subsequent sub‐theme.

##### 3.2.2.2 Subtheme 2.2: Systemic Barriers

Parents described procedural, structural and communicative barriers that limited their ability to participate meaningfully in decisions about their families. From 21 studies, these included inaccessible information, a lack of professional support, complex legal processes, administrative demands and limited reasonable adjustments. Father 16 described the difficulties of accessing benefits, ‘I filled some documents up a few times… but there was so much writing… It's a stack of papers, you know… I will not write ten pages to get or maybe not get 50 zlotys’ (Ćwirynkało and Parchomiuk 2023). Also, Toby highlighted difficulty understanding legal terminology, ‘Permanency [pause]. It is a long word I can't get out. That's what I get stuck on, the long words’ (Pytlowana and Kroese 2021). In addition, parents feel disempowered and marginalised by support systems. For example, Oksana reported ‘Child Protection came and took [the child], I was at a friend's house… and they took the child. I have no idea why… whether it's because I can't read or write or because of something else… I don't even know… they didn't say anything’. (Sumskiene and Gevorgianiene 2025). There is also a lack of support throughout and following these complex proceedings. In McCarthy (2019), a participant reported ‘After my son went, I phoned up Social Services. They didn't help me at all’. These accounts indicate when information was inaccessible, this functioned as a mechanism of exclusion: parents were placed at a disadvantage with their participation constrained in processes that had significant implications for parenting decisions and family stability.

#### 3.2.3 Theme 3: Structural Harm: Violence, Coercion and Family Separation

This analytic theme explores the escalation from judgement and control to experiences of abuse, forms of violence and coercion that can result in relational loss and structural harm. Parents described how stigma was associated with restrictions on their autonomy within families and professional systems.

##### 3.2.3.1 Subtheme 3.1: Family Conflict and Power Imbalances

This sub‐theme extended through 22 studies and captures the relational stigma enacted by family members and partners. Family members were often described as important sources of support; however, in the context of parenting, they became figures of authority and control. Parents described situations where relatives or partners restricted their autonomy over reproduction, finances, relationships or contact with children. Participants report being discouraged from parenthood and pressured to have an abortion, ‘…they thought that I should have an abortion and my brother and mother wanted me to do that, but I said that I wanted to keep the child. After that, there were no more discussions’ (Höglund and Larsson 2013). Furthermore, mothers faced challenges related to intimate partner abuse, including domestic violence during and after pregnancy and child abuse. Mothers found it difficult to speak up in these circumstances, as Paulina described, ‘He used to hit me. It went on for a long time. Until I took the child with me. That's when I ran away. I was afraid he would eventually kill me. It lasted a long time, and I said nothing. I stayed silent. I was afraid they would come and take the children away. And I was protecting them from him’. (Wołowicz and Ćwirynkało 2025). Fathers also described being excluded from family decision‐making. Father 6 stated, ‘That's all her [partner's mother] fault. She will not let come [to see a partner and a daughter] when I want… I had nothing to say’ (Ćwirynkało and Parchomiuk 2023). This highlights a significant power imbalance where the parent's adult status and autonomy are undermined.

##### 3.2.3.2 Subtheme 3.2: Child Removal, Loss and Threat

Child removal was one of the most distressing and recurrent experiences across 22 of the included studies. Parents described removal as sudden, traumatic, difficult to understand and unexpected. The threat and fear of removal shaped interactions with services; often parents were unable to regain custody, which led to attempts of reconciliation with this decision. Fran described the shock of her daughter being removed from school, ‘When she didn't come home in a taxi from school. They took her from school and they wasn't supposed to do that without asking my permission…’ (Gould and Dodd 2014). Furthermore, Michael described removal as a humiliating experience, ‘They snatched my daughter, and within about a week, it went around the pub. That they snatched her’ (Pytlowana and Kroese 2021). For some parents, removal occurred repeatedly and from birth. Wioletta stated, ‘They took away my son in the hospital. Later, the second one. The same way. They took them away in the hospital. They took away my newborn’ (Wołowicz and Ćwirynkało 2025). These accounts illustrate the difficult emotional consequences of family separation. Parents described grief, powerlessness and enduring distress, particularly where they felt that decisions were made before their parenting had been fully understood or supported. As Deirdre described, ‘…I felt worthless and I felt like I couldn't mind a child…and I wasn't a good mother. He made me feel I wasn't a good mother…’ (Sheerin et al. 2013).

##### 3.2.3.3 Subtheme 3.3: Violence, Coercion and Structural Harm

Thirteen of the included studies described parents' experiences of historical victimisation, institutional harm and coercive practices. These experiences included childhood abuse, lack of informed consent, physical abuse and professional restrictions on reproductive autonomy and intimate boundaries. Rita described violence in childhood: ‘In the school there was a teacher, who abused us a lot, beat us with a whip, a washing machine hose, I couldn't complain to anyone, if I did, I was beaten until I was blue’ (Sumskiene and Gevorgianiene 2025). Some parents' vulnerability to adult harm was linked to earlier institutional or interpersonal abuse. Sylwia reported physical abuse by staff in an institutional setting, ‘There was this place. We would kiss there and touch. It was nice. But then a caregiver came in and started yelling. I got scared and sat on the floor. He grabbed me by the clothes, right here, and pulled me out. Then he pushed my boyfriend against the wall. Threw him against the wall’. (Wołowicz and Ćwirynkało 2025). Throughout, these accounts suggest that stigma can intersect with broader experiences of violence, coercion and restricted autonomy. For some parents, institutional systems are contributing to further disempowerment.

#### 3.2.4 Theme 4: Living With Stigma: Identity, Emotional Impact and Parenting Experience

This theme explores how stigma is shaped through parents' identities, emotional distress and maintaining relationships with their children. Parents described stressful internal experiences and challenges in maintaining their parenting identities, and they also described parenting as a source of meaning, connection and self‐worth.

##### 3.2.4.1 Subtheme 4.1: Parenting Identity, Meaning and Emotional Experience

Parenting was consistently described as deeply meaningful. This sub‐theme was supported across 20 studies. For many parents, motherhood or fatherhood provided a valued identity that contrasted with deficit‐based perceptions. Alexa described motherhood, ‘Being a mother is such love that it tears me apart. Every mom, when she starts thinking how much she loves her child, bursts into tears (…) It's motherhood: love, sweat, and tears. It's worth everything’. (Wos and Baczała 2021). Lucas discussed his relationship with his children, ‘making your kids happy, makes you feel happy’ (Wilmots and Theodore 2025). Also, Emma described her child as giving her a purpose, ‘Every morning I get up, and it is, she is the reason I get up, something to look forward to…’ (Hevesi and Theodore 2024). At the same time, parents described emotional distress associated with scrutiny, judgement and separation from their children. As Gillian described, ‘Even to see a child in the High Street and I look at them and I want to cry, you know and that's really sad. Bit hard you know. Just walking around’. (Gould and Dodd 2014). The value attached to parenting meant that loss or threatened loss was particularly painful. Furthermore, stigma shaped parenting identity by undermining confidence and creating feelings of guilt and shame, while also making the parental role a source of meaning.

##### 3.2.4.2 Subtheme 4.2: Parenting Practices, Relationships and Child Experience

This sub‐theme, based on 21 papers, focuses on the immediate dyadic bond and practical interactions between parents and their children. Parents described everyday parenting practices that emphasised emotional closeness, attunement, protection and practical care. For example, feeding, playing and managing behaviour. Also, participants reflected on their parenting skills and adapting their caregiving to meet developmental changes and additional needs of their children. In conjunction with the impact of parenting self‐doubt due to experiences of stigma. Esther described the emotional bond with her children, ‘I have a bond with them. It is a unique feeling when I am around my children… I felt happy and joyful. I have this feeling in my stomach… like a goosebump’ (Hevesi and Theodore 2024). Max talked about his role as providing safety, ‘Trying to be strong for them… so they feel like they're safe and… secure at home’ (Wilmots and Theodore 2025). Fathers also described the meaning of direct caregiving. Michael recalled: ‘I used to pick her up from school every day. Take her to the park. [clears throat]’ (Pytlowana and Kroese 2021). Furthermore, Kaiden highlighted the importance of spending quality time with her child, ‘I used to take her to the museum …time just with me and her… just to keep that bond going with her’. (Wilmots and Theodore 2025). These accounts challenge assumptions that parents with intellectual disabilities lack insight into their children's needs. However, due to challenging experiences rooted in stigma, parenting self‐doubt is increased. As Becky describes the impact of judgement, ‘I couldn't take care of her at the end, I was surviving for myself. Like I tried my best, but it wasn't enough’. (Tahir and Cobigo 2025).

#### 3.2.5 Theme 5: Negotiating and Resisting Stigma Through Support and Agency

This theme focuses on how parents are active agents of their own change and highlights the importance of helpful support systems. Parents navigated and challenged stigma through relationships, advocacy, learning and demonstrating agency whilst facing significant constraints.

##### 3.2.5.1 Subtheme 5.1: Support Systems: Family, Professionals and Resources

Supportive relationships were central to parents' ability to sustain family life and manage professional systems. Informal support, particularly from extended family, partners and siblings, was often described as essential. In addition, helpful formal systems were also included, for example, social workers, peer groups and community organisations that provide practical and emotional support. Throughout 21 studies meaningful support was highlighted. Tómas described the role of his sister‐in‐law, ‘It's better to turn to the ones who are close [family]… of course there is our support worker… but Anna's sister is the rock, and she helps us raising them… the older ones stay one week with her and one week with us’ (Stefánsdóttir et al. 2023). Talia attributed her ability to retain care of her children to her husband's support, ‘They live with me now, and that is because of my husband. If my husband was not to be here, or probably was not around, my kids would have been taken away from me’. (Tahir and Cobigo 2025). Formal support was valued when it was experienced as respectful, accessible, and grounded in belief in parental capability. Vanda described a growing relationship with a social worker, ‘Well, I was afraid of her at the beginning, but she was so good, that when there was any problem, I contacted her. Then he [son] attended school without any problems, so they … don't come here anymore’. (Strnadová et al. 2019). These accounts suggest that support was most helpful when it aimed to increase parental autonomy. In addition, meaningful support to reduce isolation, increase confidence and enable parents to navigate systems more effectively.

##### 3.2.5.2 Subtheme 5.2: Advocacy, Agency and Resistance

This sub‐theme is found in 21 papers and explores parents approaches to actively resisting stigma by challenging professionals, disputing labels and learning to voice their opinions. As Marie described, ‘I speak up more for myself now than I used to… if you keep quiet that's when you get picked on’ (Franklin et al. 2022). Advocacy was valued in multiple contexts (i.e., child protection, legal proceedings) and often experienced as a way of reducing power imbalances. Parents actively negotiated intimidating professional systems with perseverance by directly challenging perceived misjudgements, for example, Madeline described her perseverance, ‘I don't give up. I am not a quitter. Yeah, I have to be (strong)…and just looking at the kids' faces kept me going’. (Hevesi and Theodore 2024). Also, Anna resisted the use of a neglect label in her child protection plan, ‘I refuse to have neglect written on the plan, I would never neglect my child’ (Stefánsdóttir et al. 2023). Parents also used strategic efforts to maintain relationships with professionals, encouraged other parents to seek advocacy, and found hope within adversity. As Maria recalled, ‘In life you go through things and they either break you or make you stronger. I've gone through too much; I didn't lose my family to gain nothing’. (Theodore et al. 2018).

##### 3.2.5.3 Subtheme 5.3: Navigating Support, Learning and Adaptation

This sub‐theme, supported by 20 papers, describes the proactive strategies parents use to identify their own support needs and engage with learning opportunities to increase their confidence. Parents described learning to accept help as a strength and utilising a range of approaches. For example, talking to others, video interaction feedback, workshops and professional guidance to gain new skills and adapt to their child's needs. Berit described the value of video feedback, ‘Everyone should try that… it's so great, so nice, you know, to know that this is where I can find support, get a bit of positive feedback’ (Starke 2010). Whilst Shazia highlighted the importance of accessible and responsive adjustments from others, ‘It's like sometimes, when she talks to me, like, I will tell her to repeat it, or she'll break it down or make it easy for me in small words’. (Atkin and Kroese 2022). In addition, participants also encouraged others to ask for help to develop parenting skills, Kelly stated: ‘Go to a social worker and say that you need help with learning difficulties… there's nothing wrong with that’ (Franklin et al. 2022). These accounts suggest that when support was collaborative, accessible, and respectful, it could enhance parental confidence and reduce the impact of stigma. Parents' willingness to learn, adapt, and seek support further challenged assumptions that they were unable or unwilling to meet their children's needs.

## 4 Discussion

This thematic synthesis identified qualitative research exploring the lived experiences of stigma among parents with intellectual disabilities. The analytic themes progressively illustrate stigma as a dynamic process within parenting experiences: constructed and enacted discursively, internalised emotionally, operationalised structurally and resisted relationally.

The first analytical theme, ‘Constructing Parents as incompetent’, illustrates how stigma is experienced through widespread assumptions of parental inadequacy. Parents described being judged because of their intellectual disability label, with incompetence often assumed without assessment‐based evidence. These experiences were frequently interpersonal and professional interactions, consistent with earlier conceptualisations of stigma (Link and Phelan 2001). Throughout the evidence base, negative public attitudes and assumptions of inability are noted (Scior 2011; Werner et al. 2012), this theme demonstrates how parents interpret and describe these perceptions across contexts (e.g., professional settings, cultures). Participants frequently described needing to demonstrate competence and emphasise responsibility, suggesting the internalisation of stigma. As such, stigma could be considered to have shaped parents' self‐perceptions and identities. This reflects broader evidence that individuals with intellectual disabilities may internalise negative societal beliefs (Logeswaran et al. 2019). Moreover, the current findings suggest that these processes may be intensified within parenting due to broader expectations of caregiving.

The second analytical theme, ‘Regulating Parenthood Through Surveillance and Control’, highlights how stigma operates within institutional systems (e.g., health and social care services). Parents described professional involvement as continuous monitoring, that was evaluative, intrusive and risk focused. These findings address how stigma is embedded within service structures and practices. Consistent with evidence that parents with intellectual disabilities are disproportionately involved in child protection systems (Booth et al. 2005; Slayter and Jensen 2019). The present synthesis extends this literature by illustrating how these processes are experienced. In addition, procedural and communicative barriers limited parents' ability to participate meaningfully in decision making. Inaccessible information and limited adjustments placed parents at a disadvantage which reinforced disempowerment. Relationships with professionals were frequently characterised by power imbalances, reflecting findings that professional decision‐making may be influenced by assumptions regarding parental competence (Proctor and Azar 2013). These findings suggest that stigma is woven through institutional processes that constrain parenting.

The third analytical theme, ‘Structural Harm: Violence, Coercion, and Family Separation’, represents an escalation of the surveillance, control and stigmatising processes outlined in Theme 2, to coercion, loss of autonomy and significant relational harm. Parents described restrictions on decision‐making within family and professional contexts, including control over reproduction, relationships and parenting practices. These experiences reflected unequal power dynamics which contributed to reduced parental autonomy. Child removal was noted as a traumatic and difficult to understand experience. Parents frequently perceived these decisions as biased toward the stereotypes associated with their diagnostic status which contributed to intense feelings (e.g., anger, grief). In addition, accounts of interpersonal (e.g., domestic abuse) and institutional violence (e.g., coercive practices) further highlight how stigma intersects with broader vulnerability and structural inequality. Fear of professional intervention often restricted engagement with services. As discussed, intellectual disability may indicate a potential of risk (Lightfoot and Slayter 2014), however, this theme highlights the impact of stigma as an embedded structure in which systems of authority facilitate decisions that contribute to harmful outcomes as experienced by parents with intellectual disabilities.

The fourth analytical theme, ‘Living with Stigma: Identity, Emotional Impact, and Parenting Experience’, captures how stigma is internalised and negotiated at an individual and relational level. Parents described emotional responses including shame, anxiety and reduced confidence, capturing the lived experience of stigma. These findings support that of adults with intellectual disabilities’ experiences of stigma and stereotyping (Jahoda et al. 2010; Logeswaran et al. 2019). The synthesis extends this literature through the specific focus on the parenting role. There is also a valued identity that provides meaning, purpose and connection with children. Descriptions of strong emotional bonds and engagement in everyday caregiving practices challenge the negative narrative that follows parents with intellectual disability. The synthesis highlights that stigma shaped parenting identity as complex and contradictory, as strong experiences of judgement and positive constructions of self as a parent existed together. This dichotomy may suggest findings of Shewan et al. (2014) are of specific relevance; of note, this paper did not meet criteria for the current review. These authors highlight that parents differentiate their identities between the label of intellectual disability and parent as separate. Therefore, parents may mediate the impact of stigma through personal meaning, relational experiences, and identity negotiation within the parenting context.

The final analytical theme, ‘Negotiating and Resisting Stigma Through Support and Agency’, identifies how parents respond to and challenge stigma. Across studies, parents demonstrated agency through advocacy, engagement with support systems, and efforts to develop parenting skills. Supportive relationships were key (i.e., family, professional and third sector organisations) to sustain family life, reduce isolation and navigate complex systems. Consistent with intervention‐focused research, support was most effective when it was collaborative, accessible and strengths‐based (Wilson et al. 2014). Parents also noted actively resisting stigma by challenging professional decisions, disputing deficit‐based labels (e.g., alleged neglect) and asserting parental competence. These findings align with research highlighting resistance among adults with intellectual disabilities (Logeswaran et al. 2019). Importantly, the processes identified suggest parental agency is facilitated through supportive relational systems.

This review highlights how stigma shapes the parenting experiences of individuals with intellectual disabilities through power imbalances, surveillance and structural inequalities, while also emphasising the value parents place on their parenting role. Across national, relational and service contexts, stigma was consistently experienced within family, professional and institutional systems, showing the transferability of the findings. Further, by offering a detailed account of how stigma is a multi‐level process of enactment, internalisation and resistance, these findings extend previous reviews. For instance, Morris et al. (2026) identified ableism as a pervasive barrier, and the present review builds on this by specifying how stigma operates across identity, professional relationships and parenting outcomes. Similarly, while Logeswaran et al. (2019) demonstrated how stigma shapes identity, the current findings show how these processes are intensified within the parenting role, highlighting the juncture between stigma, identity and socially constructed expectations of parenting competence.

### 4.1 Limitations

While all included studies were appraised as being of high methodological quality using the CASP checklist, a recurring limitation across the studies was limited reporting of reflexivity, with 10 studies providing insufficient detail regarding the researcher‐participant relationship or the influence of researcher positioning on data collection and interpretation. This limits confidence in understanding how potential power dynamics were managed across studies. A further limitation relates to sampling. Many included studies recruited through services, advocacy groups, professional networks or disability organisations, meaning that parents who were not receiving services may be underrepresented. Several studies also focused primarily on mothers, although more recent studies included fathers, partly addressing a recognised gap in the literature. Most participants were described as having mild to moderate intellectual disabilities, limiting transferability to parents with more severe or profound disabilities. The review included studies from a range of national contexts, as noted above. While this breadth strengthens the synthesis, it also introduces variation in legal, welfare and child protection systems. As a result, findings should be interpreted cautiously, as experiences of stigma, surveillance and family separation may be shaped by specific policy and cultural contexts. Finally, this review was limited to English‐language studies published between 2010 and 2025. It is therefore possible that relevant studies published in other languages or outside this timeframe were excluded. Initial coding and synthesis were primarily conducted by the first reviewer, although discussion with academic supervisors and second‐reviewer involvement in screening and quality appraisal supported rigour. Only 20% of title and abstract screening and quality appraisals were independently reviewed by a second reviewer, which may have increased potential subjectivity. All full text decisions were independently screened by two reviewers. As with qualitative syntheses, the findings represent an interpretive account rather than an exhaustive representation of all parents' experiences.

### 4.2 Clinical and Research Implications

Understanding how parents with intellectual disabilities experience stigma has important implications for social care provision and child protection practice. The findings suggest that there are barriers to services moving beyond deficit‐based assumptions. Training for professionals should include the lived experiences of parents with intellectual disabilities, particularly around judgement, monitoring, inaccessible communication and the emotional impact of child protection involvement. Such training may help professionals recognise how stigma can shape expectations of parental competence.

The review also highlights the importance of accessible strengths‐based support. Parents described support as most helpful when it promoted autonomy, reduced isolation and recognised parental capability (see Koolen et al. 2020). Clinical and social care services should therefore prioritise reasonable adjustments, accessible information, advocacy, and relationship‐based approaches that enable parents to participate meaningfully in decisions about their families. Early support may be important in reducing crisis‐led involvement and improving trust between parents and professionals.

Future research should continue to prioritise the voices of parents with intellectual disabilities, particularly those who are less visible in current research, including fathers, parents with more severe intellectual disabilities, parents from minority ethnic communities, and parents not connected to services or advocacy groups. Further research is also needed to explore how stigma operates across different cultural, legal and child protection contexts, and to evaluate interventions designed to reduce stigma, improve professional practice, and support parents' rights, identity and family relationships. These data support the value of directly listening to the perspective of parents on their lived experiences of stigma; it is crucial that these identified needs inform service development and professional practice toward respectful and supportive approaches which promote autonomy.

## Funding

The authors have nothing to report.

## Conflicts of Interest

The authors declare no conflicts of interest.

## Data Availability

Data sharing not applicable to this article as no datasets were generated or analysed during the current study.
